# Differences in Pain Management Strategies Between Mobile and Immobile Hospitalized Patients: A Real‐World Data Analysis

**DOI:** 10.1155/prm/5597200

**Published:** 2026-02-17

**Authors:** Juli Thomaz de Souza, Thaís Caroline da Silva Piccoli, Victória Moralez Soares, Manuela Hoedl

**Affiliations:** ^1^ Department of Internal Medicine, São Paulo State University (UNESP), Botucatu Medical School, Botucatu/SP, Brazil, unesp.br; ^2^ Institute of Nursing Science, Medical University of Graz, Neue Stiftingtalstraße 6/P06–WEST, Graz, 8010, Austria, medunigraz.at

**Keywords:** mobility limitation, pain, pain management

## Abstract

**Background:**

Hospital‐associated deconditioning is complex and multifactorial and has been shown to be closely linked to immobility, which, in turn, has serious consequences. Additionally, pain is seen as one major contributing factor impeding mobility and, therefore, increasing immobility. This study aimed to compare pain management interventions between mobile and immobile patients.

**Design:**

A cross‐sectional study using real‐world data focusing on patients in Austrian hospitals.

**Methods:**

Data were collected by trained nurses in three periods (2021–2023). Patients were classified as mobile or immobile based on the mobility subscale of the Braden Scale. Statistical analysis involved, e.g., chi‐square and Mann–Whitney tests, with *p* < 0.05 being considered significant.

**Results:**

A total of 3214 patients had pain, of which 1661 were mobile and 1553 were immobile. Immobile patients were statistically significantly older, had more comorbidities, and were also more care dependent than mobile patients, measured with the Care Dependency Scale. Regarding pain management statistically significant, associations can be found between mobile and immobile patients regarding physiotherapy (24.7 vs. 53.1), occupational therapy (6.4 vs. 9.2), and pharmacological treatments (79.5 vs. 91.6), while mobile patients showed associations with alternative therapies such as acupuncture (1.1. vs. 0.3) and relaxing therapies (3.9 vs. 2.3).

**Conclusion:**

Our study showed that immobile patients were associated with conventional interventions, while mobile patients showed an association with alternative therapies. In order to reduce these disparities, increasing the awareness of healthcare professionals in order to personalized pain management strategies is needed. Future research, especially qualitative or mixed‐method studies, focusing on the decision making of mobile and immobile patients and including other pain specific aspects such as pain intensity or location is also recommended.

**Summary**
• Reporting Method
◦ The STrengthening the Reporting of OBservational studies in Epidemiology (STROBE) checklist.
• Patient or public contribution
◦ There is no patient or public contribution.
• What does this paper contribute to the wider global clinical community?
◦ Immobile patients and mobile patients were associated with different pain therapies
◦ Awareness of offering alternative treatment options to immobile patients should be increased.
◦ Qualitative studies, focusing on the decision making of mobile and immobile patients are needed.

## 1. Introduction

Hospitalization is seen as a stressful event for the patient. In a recent systematic review and meta‐analysis, the authors showed that patients are exposed to high levels of stress during hospital stay [1]. High levels of stress are statistically significantly associated with patient outcomes such as patient satisfaction, length of stay, or pain [1]. Moreover, such high level of stress might even lead to adverse events after discharge [1], an accelerated loss of independence, or functional decline [2].

This negative consequence of hospitalization is called hospital‐associated deconditioning [3]. Authors of a systematic review and meta‐analysis concluded that it may result in delayed discharge and an increased likelihood of admission or readmission to, e.g., a nursing home [3]. Hospital‐associated deconditioning also increases healthcare costs.

The American Hospital Association estimates that hospital‐associated deconditioning was responsible for costs of about 58.5 billion US dollars in 2019, which accounts for 8.3% of the year’s total annual medical spending in the United States of America [4]. Even though hospital‐associated deconditioning is complex and multifactorial, several studies state bed rest or prolonged periods of immobility as one of the main factors [2, 3, 5].

Two studies reported that patients spent an average of 83% of their time in bed, 12% in a chair, and only 30 min a day being physically active [6, 7]. This is similar to the results of three other studies which described that hospital patients spend more than 95% of their time in a bed or chair [8–10]. Another study reported that the average duration of immobility in stroke patients ranges from 7.36 up to 12.11 days [11]. Hence, immobility seems to be still the current status in hospitals, with far‐reaching consequences [12].

A multitude of adverse patient outcomes are linked to immobility [13]. The authors of one study concluded that immobility for more than 3 days is a risk factor for proximal deep vein thrombosis in acutely ill medical inpatients [14]. Another team of researchers found that 54% of their 22 immobile patients were at risk of malnutrition or malnourished [15]. These observations lead them to conclude that 2 weeks of disease‐related immobilization result in a significant loss of thigh muscle mass and muscle strength in older patients with impaired mobility [15]. Another study found that prolonged bed rest can lead to an increased tendency to fall when patients were mobilized the first time [2].

Moreover, immobile patients have a higher risk of urinary tract infection [16] as well as a higher total complication rate regarding immobility, incidence of pneumonia, and incidence of pressure sore development [17, 18]. Immobility does not only affect the patients but also the healthcare systems in terms of costs. For the Chinese healthcare system, the median costs for immobile stroke patients ranged between RMB 47000.68 and RMB 16578.44 [11].

There are several factors which contribute to immobility during hospitalization, such as the hospital environment or a hospital culture and processes favoring immobility [19, 20]. Two studies investigated immobility from the staff perspective. The first study investigated the use of systematic functional measurements to combat negative effects of immobility in the acute setting. In the second study, the authors found that patients with pain are more challenging in terms of promoting mobility [12]. Another study reported that pain is one of the issues hindering mobility because of the side effects of pain medication and the pain itself, which makes it challenging to mobilize the affected patients [2].

Other reasons for immobility are patient related, such as surgical trauma, infections, mental stress, changes in food intake, or pain [15]. Two interview studies with patients highlighted weakness, lethargy, shortness of breath, dizziness, nausea, body stiffness, and pain as the main factors affecting their ability to walk independently during hospitalization [21, 22].

It can, therefore, be concluded that hospital‐associated deconditioning is complex and multifactorial and negatively affects patients and the healthcare system. Hospital‐associated deconditioning has been shown to be closely linked to immobility, which, in turn, has serious consequences. In addition, patients as well as healthcare staff highlighted pain as one major contributing factor impeding mobility and, therefore, increasing immobility. But if pain is a main cause of immobility, leading to hospital‐associated deconditioning, the question remains which interventions are performed to manage pain in mobile and immobile patients.

The present study was designed to address these gaps by comparing pain management interventions in a large sample of mobile versus immobile patient. Determining the performed pain management interventions in mobile versus immobile patient can help to identify gaps in the pain management in both groups, restructure pain management protocols, which in turn can improve satisfaction and outcomes in mobile and immobile patients.

## 2. Methods

### 2.1. Study Design, Setting, and Participants

This is a cross‐sectional study with real‐world data from the “International Prevalence Measurement of Care Problems” study, which investigates various healthcare problems such as malnutrition, falls, and pain [23–25]. For the present study, we only analyzed data related to pain. All Austrian hospitals with more than 50 beds are invited to participate in the study. Participants were considered patients if they had experienced pain in the previous 7 days and were divided into two groups: mobile patients and immobile patients.

### 2.2. Data Collection Process

The coordinators of the hospitals took part in a training session which was organized by the research team, during which the standardized data collection procedure and the questionnaire, as well as the data‐entry program were explained in detail and training materials were handed out.

A team of two nurses, one of whom works on the ward being surveyed and the other one on a different ward, collected the data on November 10–12, 2021, November 09–11, 2022, and November 08–10, 2023. Both nurses assessed each patient independently either at bedside (e.g., degree of care dependency and pain frequency) or by means of medical records, such as pain interventions. In case of disagreement between the two nurses, the decision from the nurse from the “other” ward was chosen to enhance objectivity. After the data collection, the nurses, who also had a training for the data‐entry program, entered the data into the data‐entry program.

### 2.3. Data Collection Instrument

In this study, we used the Austrian version of the International Prevalence Measurement of Care Problems in Care Homes [23–25]. This questionnaire was developed by an expert team based on evidence‐based guidelines and expert consensus and was found to be a valid and reliable instrument. It is also updated regularly.

Demographic data and patients’ medical diagnoses according to the ICD 10 [26] were collected from the medical records. Furthermore, the team of two nurses assessed the degree of each patient’s care dependency by using the German version of the Care Dependency Scale (CDS) [26, 27]. By means of 15 items, e.g., eating and drinking, continence, or body posture [26, 27], each patient was rated from completely dependent (1 point) up to almost care independent (5 points).

The total sum scores of the scale were categorized as follows: 15–24, completely care dependent; 25–44, to a great extent care dependent; 45–59, partially care dependent; 60–69, to a limited extent care dependent; and 70–75, almost care independent.

In order to be able to distinguish between mobile and immobile patients, we used the subitem mobility of the Braden Scale [28], which is a valid and reliable instrument [29, 30]. The subitem mobility is defined “the client’s ability to change or control their body position” [28]. For the purpose of this study, slightly (makes frequent though slight changes in body or extremity position independently), very immobile (makes occasional slight changes in body or extremity position but unable to make frequent or significant changes independently), and completely immobile patients (does not make even slight changes in body or extremity position without assistance) were classified as immobile patients. Whereas patients without any mobility limitations (makes major and frequent changes in position without assistance) were characterized as mobile patients.

With regard to pain, all patients were asked whether they had suffered pain during the previous 7 days. Patients with daily pain or isolated pain experiences were characterized as patients. The pain management interventions on the questionnaire included physiotherapy, occupational therapy, or pharmacological treatment (possible answers: yes or no).

### 2.4. Data Analysis

To calculate the differences between the mobile and immobile patients, a chi‐square test was performed to evaluate categorical variables, and the results were expressed as numbers and percentages. For categorical variables’ effect size, Phi including the *p* value of the effect size was calculated. Additionally, the confidence interval for the Phi was calculated using the effect size calculator for a chi‐square based on a 2‐by‐2 frequency table [31].

To evaluate the difference between the groups concerning patient age, a Mann–Whitney *U* test as well as Spearman for effect size was performed, and the result was expressed as the median and 25th‐75th percentiles. A *p* value of < 0.05 indicates statistical significance. A Bonferroni correction, as recommended by Field (2024) was applied using all 25 interventions in the model (Table 1), resulting in statistical significance being accepted when *p* < 0.002 [34]. The IBM SPSS program (Version 25.0. Armonk, NY: IBM Corp) was used.

**TABLE 1:** Pain management interventions in mobile and immobile patients.

	**Mobile (*N* = 1661)**	**Immobile (*N* = 1553)**	**p**	**Effect size**	**CI for effect size**	**p** **for effect size**

Pharmacological interventions, *n* (%)	1320 (79.5)	1422 (91.6)	*p* ≤ 0.001	0.171	0.137–0.204	*p* ≤ 0.001
Nonopioid analgesics, *n* (%)	1260 (75.9)	1280 (82.4)	*p* ≤ 0.001	0.081	0.046–0.115	*p* ≤ 0.001
Nonpharmacological treatments, *n* (%)	808 (48.6)	1034 (66.6)	*p* ≤ 0.001	0.181	0.147–0.214	*p* ≤ 0.001
Physiotherapy, *n* (%)	411 (24.7)	824 (53.1)	*p* ≤ 0.001	0.291	0.259–0.322	*p* ≤ 0.001
Opioid analgesics, *n* (%)	343 (20.7)	734 (47.3)	*p* ≤ 0.001	0.282	0.249–0.313	*p* ≤ 0.001
Strong‐acting opioids, *n* (%)	307 (18.5)	671 (43.2)	*p* ≤ 0.001	0.269	0.236–0.300	*p* ≤ 0.001
NSAIDs, *n* (%)	649 (39.1)	579 (37.3)	*p* = 0.309	−0.018	−0.017–0.052	*p* = 0.297
Paracetamol, *n* (%)	420 (25.3)	480 (30.9)	*p* ≤ 0.001	0.063	0.026–0.095	*p* ≤ 0.001
Other nonopioid analgesics, *n* (%)	415 (25.0)	468 (30.1)	*p* ≤ 0.001	0.058	0.021–0.090	*p* ≤ 0.001
Patient education, *n* (%)	339 (20.4)	417 (26.9)	*p* ≤ 0.001	0.076	0.040–0.109	*p* ≤ 0.001
Cold/heat therapy, *n* (%)	168 (10.1)	208 (13.4)	*p* ≤ 0.05	0.051	0.015–0.084	*p* ≤ 0.05
Other nonpharmacological interventions, *n* (%)	158 (9.5)	179 (11.5)	*p* = 0.065	0.033	−0.004‐0.065	*p* = 0.063
Immobilization and bracing, *n* (%)	111 (6.7)	170 (10.9)	*p* ≤ 0.001	0.075	0.040–0.109	*p* ≤ 0.001
No interventions, *n* (%)	159 (9.6)	52 (3.3)	*p* ≤ 0.001	−0.126	−0.090‐0.159	*p* ≤ 0.001
Antidepressants, *n* (%)	96 (5.8)	147 (9.5)	*p* ≤ 0.001	0.070	0.036–0.105	*p* ≤ 0.001
Occupational therapy, *n* (%)	106 (6.4)	143 (9.2)	*p* ≤ 0.05	0.053	0.018–0.087	*p* ≤ 0.05
Antiepileptics, *n* (%)	97 (5.8)	121 (7.8)	*p* ≤ 0.05	0.039	0.000–0.070	*p* ≤ 0.05
Other interventions, *n* (%)	98 (5.9)	72 (4.6)	*p* = 0.115	−0.028	−0.009‐0.060	*p* = 0.110
Weak‐acting opioids, *n* (%)	40 (2.4)	76 (4.9)	*p* ≤ 0.001	0.067	0.032–0.100	*p* ≤ 0.001
Psychobehavioral therapy, *n* (%)	89 (5.4)	75 (4.8)	*p* = 0.522	−0.012	−0.035‐0.035	*p* = 0.496
Relaxing therapies, *n* (%)	65 (3.9)	36 (2.3)	*p* ≤ 0.05	−0.046	−0.009‐0.078	*p* ≤ 0.05
Music therapy, *n* (%)	19 (1.1)	43 (2.8)	*p* ≤ 0.001	0.059	0.024–0.093	*p* ≤ 0.001
Acupuncture, *n* (%)	18 (1.1)	5 (0.3)	*p* ≤ 0.05	−0.045	−0.009‐0.078	*p* ≤ 0.05
Patient refused pain interventions, *n* (%)	10 (0.6)	5 (0.3)	*p* = 0.305	−0.021	−0.017‐0.052	*p* = 0.244
TENS, *n* (%)	7 (0.4)	8 (0.5)	*p* = 0.798	0.007	−0.035‐0.035	*p* = 0.697

*Note:* Other nonpharmacological interventions are other nonpharmacological interventions that are not mentioned in the list; Relaxing therapies are, e.g., yoga and mindfulness. The results were expressed as numbers and percentages. Chi‐square/Phi was performed to evaluate categorical variables; Bonferroni correction *p* < 0.002.

Abbreviations: NSAIDs, nonsteroidal anti‐inflammatory drugs; TENS, transcutaneous electrical nerve stimulation.

### 2.5. Ethics

We adhered to the Code of Ethics of the World Medical Association (Declaration of Helsinki). The ethical committee of the Medical University of Graz gave their ethical approval for each year of data collection (number: 20‐192 ex 08/09; 04. March 2021/03.June. 2022/12. May 2023). Patients were invited to participate if they were present at the measurement days in the hospital. A written informed consent was obtained from each patient or their legal representatives.

## 3. Results

### 3.1. Recruitment and Participants’ Characteristics

**FIGURE 1:**
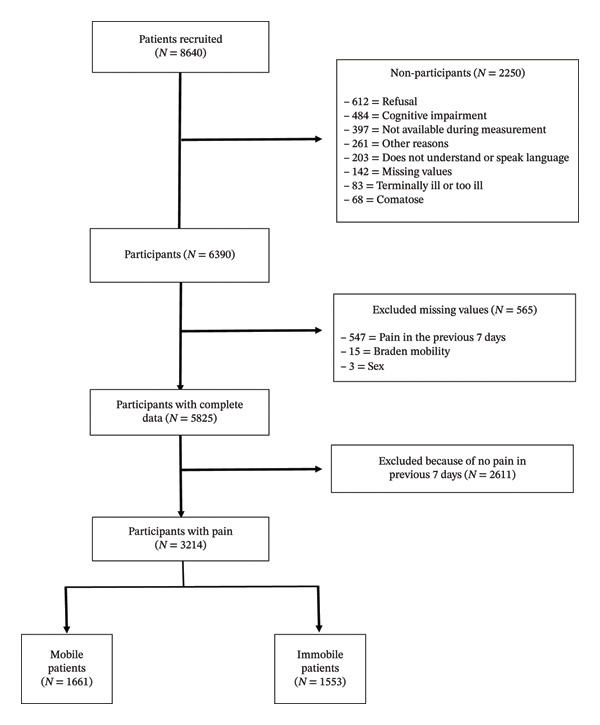
Flowchart of study participant inclusion.

A total of 8640 patients were recruited and 5825 were included in the study, of which 3214 patients reported having suffered pain. The flowchart of patient recruitment and inclusion is shown in Figure 1.

Regarding the general characteristics of the participants, immobile patients were older and had more comorbidities than mobile patients. The general characteristics of mobile and immobile patients are shown in Table 2.

**TABLE 2:** General characteristics of mobile and immobile patients.

	**Mobile (*N* = 1661)**	**Immobile (*N* = 1553)**	**p**	**Effect size**	**p** **for effect size**

Female sex, *n* (%)	848 (51.1)	843 (54.3)	*p* = 0.071	0.032	*p* = 0.067
Age (years)	63 (50–75)	75 (65–83)	*p* ≤ 0.001	0.349	*p* ≤ 0.001

Common diagnoses, n (%)					
Cardiovascular diseases	588 (35.4)	753 (48.5)	*p* ≤ 0.001	0.133	*p* ≤ 0.001
Cancer/neoplasms	367 (22.1)	366 (23.6)	*p* = 0.333	0.018	*p* = 0.320
Diseases of the digestive system	337 (20.3)	293 (18.9)	*p* = 0.310	−0.018	*p* = 0.310
Diseases of the musculoskeletal system	328 (19.7)	454 (29.2)	*p* ≤ 0.001	0.110	*p* ≤ 0.001
Diseases of the respiratory system	284 (17.1)	378 (24.3)	*p* ≤ 0.001	0.089	*p* ≤ 0.001
Diseases of the genitourinary system	254 (15.3)	340 (21.9)	*p* ≤ 0.001	0.085	*p* ≤ 0.001
Diabetes mellitus	211 (12.7)	318 (20.5)	*p* ≤ 0.001	0.105	*p* ≤ 0.001
Infectious and parasitic diseases	101 (6.1)	175 (11.3)	*p* ≤ 0.001	0.093	*p* ≤ 0.001
Stroke	42 (2.5)	94 (6.1)	*p* ≤ 0.001	0.087	*p* ≤ 0.001
Dementia	16 (1.0)	83 (5.3)	*p* ≤ 0.001	0.127	*p* ≤ 0.001

*Note:* Chi‐square/Phi was performed to evaluate categorical variables; the results were expressed as numbers and percentages. Mann–Whitney U/Spearman was performed for age; the result was expressed as the median and 25th–75th percentiles.

### 3.2. Degree of Care Dependency

With regard to the CDS sum scores, immobile patients (*n* = 1553) were statistically significantly more dependent (median = 60 [46–69]) than mobile patients (*n* = 1661) with a median care dependency of 75 (74–75). Thus, immobile patients were to a limited extent care dependent, whereas mobile patients were almost care independent.

**FIGURE 2:**
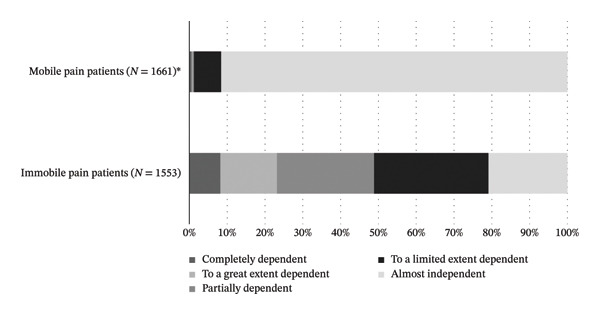
Care dependency categories in mobile and immobile patients according to the Care Dependency Scale sum scores.

Of the 1661 mobile patients, 10 (0.6%) were completely care dependent, 4 (0.2%) were to a great extent care dependent, 7 (0.4%) were partially care dependent, 119 (7.2%) were almost care independent, and 1521 (91.6%) were completely care independent. Of the 1553 immobile patients, 127 (8.2%) were completely care dependent, 233 (15%) were to a great extent care dependent, 399 (25.7%) were partially care dependent, 470 (30.3%) were almost care independent, and 324 (20.9%) were completely care independent, which constitutes a statistically significant difference between the two groups (Figure 2).

**FIGURE 3:**
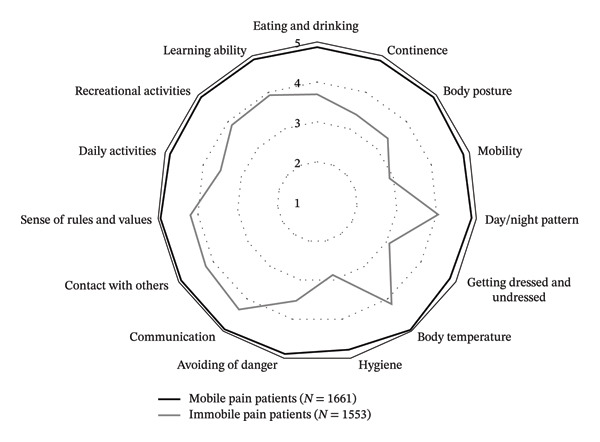
Average degree of care dependency among mobile and immobile patients according to the Care Dependency Scale.

To obtain a detailed insight to the degree of care dependency of mobile versus immobile patients, we also calculated the mean for each item; these are shown in Figure 3. Immobile patients were more care dependent for each item, and particularly so with regard to the items of mobility and getting dressed and undressed as well as hygiene.

### 3.3. Pain Management Interventions

With respect to pain management, statistically significant (*p* < 0.05) associations can be found between immobile and mobile patients regarding physical therapy, occupational therapy, cold/heat therapy, and TENS therapy, as well as other nonpharmacological and pharmacological interventions. On the other hand, mobile patients showed associations with other therapies like acupuncture and relaxing therapies, such as yoga or mindfulness training than immobile patients. Cold/heat therapy and occupational and relaxing therapies as well as acupuncture were not statistically significant after the Bonferroni correction. This could be due to the fact that all of these interventions were seldom used in both groups.

The used pain management interventions for the mobile and immobile patients are shown in Table 1.

## 4. Discussion

This real‐world data‐based study aimed to compare pain management interventions in mobile versus immobile patients. Our findings showed that immobile patients are older and have a higher prevalence of cardiovascular and respiratory diseases as well as a higher degree of care dependence, suggesting that in addition to pain, other factors may increase these patients’ functional incapacity. Furthermore, differences in therapeutic approaches were observed: While immobile patients were associated more frequently with physiotherapy, occupational therapy, and drug and nondrug treatments, mobile patients tend to use alternative therapies such as acupuncture, relaxation, yoga, and mindfulness training. We want to mention here that we did not collect data on either these alternative therapies occurred in‐hospital or external.

Immobile patients were older than mobile patients and had more comorbidities. This may be explained by the impact that aging itself has on an individual: With advancing age, physiological, biochemical, morphological, and psychosocial changes can lead to functional decline and greater susceptibility to diseases [32]. Furthermore, bedridden older individuals experience a greater loss of muscle mass, strength, and function, which, in turn, increases the time spent in bed [33, 34]. Therefore, the considerable age and care dependency difference between the mobile and immobile groups may also act as a confounding variable in treatment comparisons.

However, it is common for individuals with reduced mobility or with immobility, especially hospitalized adults and older people, to experience situations that cause pain, such as invasive procedures, fractures, back pain due to bed rest, chronic pain before hospitalization, joint pain, and neuropathic pain. This may directly impact the quality of life of these individuals, which is why adherence to pain treatment is essential in these situations [35].

In this study, it was observed that mobile patients were associated with alternative treatments more than those with mobility problems. Mobile patients’ access to alternative therapies may be related to their socioeconomic conditions, as these are generally not included in most public health services. Perhaps immobile patients have more difficulties finding alternative therapies and end up choosing or accepting the therapeutic decisions that are available according to their conditions. However, we cannot state with certainty that this may be the reason for the observed difference in patients’ treatments. Another explanation might be that even though these alternative therapies such as patient education or cold/heat treatment belonging in Austria to the field of nursing practice and, therefore, should be offered in Austrian hospitals. However, with regard to the worldwide nursing staff shortage, which is also true for Austria, alternative therapies might be the first interventions not offered.

We want to highlight that there are several reasons why a patient with or without mobility impairments may choose a certain treatment option as the most appropriate one for their situation. These decisions may be influenced by socioeconomic issues, acceptance of the disease itself, polypharmacy, and side effects of medications. In addition, the cognitive capacity for decision‐making, the assistance of a multidisciplinary team, the person’s family support network and health service providers as well as their access to various types of treatment can all influence an individual’s treatment choice [36].

It is known that a patient’s autonomy in choosing their treatment is not always guaranteed, as, in some cases, this person is unable to act autonomously and needs somebody else to represent them [37].

As this is a study with real‐world data, some limitations have to be mentioned. The first one is the use of the Braden Scale as classification into mobile and immobile patients. The Braden Scale was developed for pressure‐injury risk and not comprehensive functional mobility. This may raise misclassification concerns, as patients with assistive devices may be “mobile” in terms of physical therapy but “limited” on Braden Scale. However, this study gives a first insight into the differences in pain management between mobile and immobile pain patients, which can also be seen as a strength.

Second, the lack of more complete information on treatment adherence or reasons for patients’ therapeutic choices might be a limitation. Additionally, the big age difference may be a limitation on the one hand. This considerable age and care dependency difference between the mobile and immobile groups likely acts as a confounding variable in treatment comparisons and might, therefore, could probably explain the differences in uptake of particular nonpharmacological interventions. Furthermore, this manuscript is an outcome of a scholarship of MH as a visiting collaborating professor at São Paulo State University (UNESP), Botucatu Medical School, Brazil. So, going deeper into analyses is beyond the scope of this paper.

Third, we had no information about whether these choices were made by the patients themselves or by their guardians/caregivers/assisting team. Also, the data did not show exactly the type, location, and intensity of the pain suffered by the respective patients. Above that, we want to mention that this study was conducted solely in Austrian hospitals. So, generalizability may be decreased for countries with other healthcare service structures. However, the high sample size in mobile and immobile patients has to be highlighted as a strength.

## 5. Conclusion

In summary, immobile patients are more care dependent and were associated with conventional pain treatments more often than mobile patients, while mobile patients tend toward alternative therapies more than immobile patients. This highlights the need for support from healthcare professionals to make alternative treatment options also more accessible to immobile patients.

It is, therefore, strongly recommended that all patients have equitable access to comprehensive and high‐quality pain management. Additionally, personalized care strategies are needed to improve pain management and clinical outcomes. Lastly, future research, especially qualitative or mixed‐method studies, focusing on the decision making of mobile and immobile patients and including other pain specific aspects such as pain intensity or location is strongly recommended [26, 34].

## Author Contributions

Substantial contributions to conception and design, or acquisition of data, or analysis and interpretation of data: Juli Thomaz de Souza, Thaís Caroline da Silva Piccoli, Victória Moralez Soares, and Manuela Hoedl.

Drafting the article or revising it critically for important intellectual content: Juli Thomaz de Souza, Thaís Caroline da Silva Piccoli, Victória Moralez Soares, and Manuela Hoedl.

Final approval of the version to be published: Juli Thomaz de Souza, Thaís Caroline da Silva Piccoli, Victória Moralez Soares, and Manuela Hoedl.

All authors contributed significantly to the work.

## Funding

This article is the outcome of a scholarship of Manuela Hoedl as a visiting collaborating professor at São Paulo State University (UNESP), Botucatu Medical School, Brazil. This scholarship was funded by Brazilian Federal Agency for Support and Evaluation of Graduate Education (CAPES) (88887.979229/2024‐00). Open access funding was provided by Medizinische Universitat Graz/KEMÖ.

## Disclosure

There is a statistician on the author team (MH). The authors agree to take responsibility for ensuring that the choice of statistical approach is appropriate and is conducted and interpreted correctly as a condition to submit to the Journal.

## Ethics Statement

The ethical committee of the Medical University of Graz gave their ethical approval for each year. The latest day of approval was 12 May 2023, with the number 20‐192 ex 08/09.

## Consent

A written informed consent was obtained from each patient or their legal representatives.

## Conflicts of Interest

The authors declare no conflicts of interest.

## Data Availability

The data that support the findings of this study are available from the corresponding author upon reasonable request.
